# APOE4 enhances age-dependent decline in cognitive function by down-regulating an NMDA receptor pathway in EFAD-Tg mice

**DOI:** 10.1186/s13024-015-0002-2

**Published:** 2015-03-05

**Authors:** De-shan Liu, Xiao-dong Pan, Jing Zhang, Hui Shen, Nicole C Collins, Arron M Cole, Kevin P Koster, Manel Ben Aissa, Xiao-man Dai, Meng Zhou, Leon M Tai, Yuan-gui Zhu, Mary Jo LaDu, Xiao-chun Chen

**Affiliations:** Department of Neurology, Fujian Medical University Union Hospital, 29 Xinquan Road, 350001 Fuzhou, China; Fujian Institute of Geriatrics, Fujian Medical University Union Hospital, 29 Xinquan Road, 350001 Fuzhou, China; Key Laboratory of Brain Aging and Neurodegenerative Disease, Fujian Key Laboratory of Molecular Neurology, Fujian Medical University, 29 Xinquan Road, 350001 Fuzhou, China; Department of Anatomy and Cell Biology, University of Illinois at Chicago, 808 S.Wood St., M/C 512, 60612 Chicago, IL USA

**Keywords:** Alzheimer’s disease, Apolipoprotein E, Behavior, Synaptic proteins, Signaling pathways, Transgenic mice

## Abstract

**Background:**

Alzheimer’s disease (AD) causes progressive loss of memory and cognition, exacerbated by *APOE4,* the greatest genetic risk factor for AD. One proposed mechanism for apolipoprotein E (apoE) effects on cognition is via NMDAR-dependent signaling. *APOE* genotype-specific effects on this pathway were dissected using EFAD-transgenic (Tg) mice (5xFAD mice, that over-express human amyloid-beta (Aβ) via 5 familial-AD (FAD) mutations, and express human apoE), and 5xFAD/*APOE*-knockout (KO) mice. Previous data from EFAD-Tg mice demonstrate age-dependent (2-6 months), apoE-specific effects on the development of Aβ pathology. This study tests the hypothesis that apoE4 impairs cognition via modulation of NMDAR-dependent signaling, specifically via a loss of function by comparison of E4FAD mice with 5xFAD/*APOE*-KO mice, E3FAD and E2FAD mice.

**Results:**

Using female E2FAD, E3FAD, E4FAD and 5xFAD/*APOE*-KO mice aged 2-, 4-, and 6-months, the Y-maze and Morris water maze behavioral tests were combined with synaptic protein levels as markers of synaptic viability. The results demonstrate a greater age-induced deficit in cognition and reduction in PSD95, drebrin and NMDAR subunits in the E4FAD and 5xFAD/*APOE*-KO mice compared with E2FAD and E3FAD mice, consistent with an apoE4 loss of function. Interestingly, for NMDAR-mediated signaling, the levels of p-CaMK-II followed this same apoE-specific pattern as cognition, while the levels of p-CREB and BDNF demonstrate an apoE4 toxic gain of function: E2FAD > E3FAD > 5xFAD/*APOE*-KO > E4FAD.

**Conclusion:**

These findings suggest that compared with E2FAD and E3FAD, E4FAD and 5xFAD/*APOE*-KO mice exhibit enhanced age-induced reductions in cognition and key synaptic proteins via down-regulation of an NMDAR signaling pathway, consistent with an apoE4 loss of function. However, levels of p-CREB and BDNF, signaling factors common to multiple pathways, suggest a gain of toxic function. Publications in this field present contradictory results as to whether *APOE4* imparts a loss or gain of function. As with the results reported herein, the overall effect of *APOE4* on a given CNS-specific measure will be the product of multiple overlapping mechanisms. Thus, caution remains critical in determining whether *APOE* gene inactivation or therapies that correct the loss of positive function related to apoE4, are the appropriate therapeutic response.

**Electronic supplementary material:**

The online version of this article (doi:10.1186/s13024-015-0002-2) contains supplementary material, which is available to authorized users.

## Introduction

Alzheimer’s disease (AD) is a progressive neurodegenerative disease that causes loss of memory and cognitive function, and is the most common cause of dementia in individuals over the age of 60. *APOE4,* the greatest genetic risk factor for sporadic Alzheimer’s disease (AD), increases risk ~3- and 15-fold with a single or double allele [1-11] compared to *APOE3*, whereas *APOE2* decreases AD risk ~2-fold per allele [12-16]. The multifactorial mechanisms through which apolipoprotein E (apoE) affects AD risk ultimately converge on modulation of cognitive function. As well, the amyloid-β peptide (Aβ) [17-19], the proposed proximal neurotoxin in AD, is a major cause of impaired synaptic function, particularly soluble oligomeric forms of the peptide (oAβ) [20-23]. However, how human (h)-apoE interacts with Aβ to affect cognitive function, and the potential underlying neuronal signaling pathways, remains unclear, in part due to the lack of a tractable familial AD (FAD)-Tg mouse model. In addition, debate continues on whether apoE4 represents an overall loss of positive function or gain of toxic function, a distinction that significantly impacts therapeutic approaches for targeting not only *APOE4*-induced AD risk, but for the effects on all h-*APOE* genotypes.

In AD patients, *APOE4* is associated with an earlier age of onset for cognitive deficits than *APOE3* [6-11], and possibly a faster rate of cognitive decline [24,25], though results are conflicting regarding the latter. However, even in the absence of AD, older *APOE4* carriers (60+ years of age) exhibit deficits in episodic memory and higher rates of cognitive decline compared to *APOE3* carriers [26-29]. Although these data demonstrate greater apoE4-induced cognitive impairment compared to apoE3, it remains unclear whether this is a loss of positive function or gain of toxic function. This issue is highlighted by a recent case report of a 40-year-old male patient with an ablative frame shift mutation that results in a complete lack of apoE [30]. The patient is described as cognitively normal on gross functional tests (MMSE), raising the hypothesis that all the h-*APOE* genotypes are either a gain of toxic function, or are not required for cognitive function. However, sub-domain tests indicate deficits in memory, language, visual-spatial abilities and executive function, in addition to signs of dyslexia [30], supporting the loss of function hypothesis. Data from Tg mouse models on the role of apoE on cognitive decline are primarily derived from models that express h-apoE*,* but without h-Aβ pathology. As with non-AD patients, in *APOE-*TR mice, apoE4 is associated with cognitive deficits in both young (Morris water maze, Barnes maze) [31,32] and older mice (Morris water maze, Y-maze) [33,34]. Similar data were also observed in mice expressing h-apoE under the control of heterologous promoters (reviewed [35]). In FAD-Tg mouse models expressing h-apoE under the control of the NSE promoter, behavioral performances (water maze) follow the pattern apoE3 *>* apoE4 = apoE-knockout (KO), consistent with a loss of positive function for apoE4 [36]. However, as apoE is physiologically expressed by glia, the relevance of these data is unclear.

At the synaptic level, AD patients exhibit decreased levels of postsynaptic intracellular scaffold proteins, including postsynaptic density protein 95 (PSD95) and drebrin, suggesting post-synaptic disruption precedes loss of pre-synaptic proteins to initiate the cognitive deficits characteristic of the disease (reviewed in [37-39]). Importantly, decreased levels of PSD95 and drebrin can lead to decreased expression of *N*-methyl-D-aspartate receptor (NMDAR) subunits (N1, NR2A and NR2B) [37,38]. Clinically, *in vivo* and *in vitro* evidence indicate that AD, Aβ, inflammation and chronic vasculitis can result in chronic NMDAR activation, disrupting postsynaptic ionic gradients, long-term potentiation (LTP) and cognition [37-39]. Further, lower NDMA receptor levels may result in a decreased Ca^2+^-dependent activation of the calcium-calmodulin-II (CaMK-II)/cAMP response binding element peptide (CREB) pathway, leading to decreased production of the brain derived neurotropic factor (BDNF), critical for synaptic function and for increasing NMDAR levels via positive feedback [39-43]. Mechanistically, an apoE4-induced reduction in post-synaptic proteins may disrupt CaMK-II/CREB/BDNF signaling to impair cognitive function [44]. Similar effects are observed in long-term primary neuron-glia co-cultures, as apoE4 accelerates the loss of GluN1 levels and mature spines compared to apoE3 [45]. Further, by inducing intracellular sequestration, apoE4 reduces neuronal cell-surface expression of NMDA receptors *in vitro* [46]. However, little is known about the *APOE* genotype-specific effects on these processes in combination with AD pathology.

To assess whether apoE4 imparts a loss or gain of function requires a comparison to the absence of apoE (*APOE*-KO), not simply a comparison to apoE2/apoE3. For example, *in vivo* studies demonstrate that with LPS-induced inflammation and amyloid deposition, apoE4 is anti-inflammatory [47] and anti-amyloidigenic [35,48] compared to apoE-KO, though apoE3 is better than apoE4. In other data more directly related to synaptic dysfunction, no differences were observed between apoE4 and apoE-KO in measures including spine density and LTP [49,50], with apoE3 higher than both. Finally, apoE4 exhibits a gain of toxic function compared to apoE-KO for oAβ42-dependent attenuation of LTP [51] and oAβ42-induced neurotoxicity in neuron/glial co-cultures [52]. Thus, it is critical to determine the effect of h-apoE on postsynaptic protein expression and signaling in the EFAD and 5xFAD/*APOE*-KO mice.

As data indicate that the *APOE4*-induced risk for AD is significantly greater in females compared to males in both humans and *APOE*-TR mice [35,53-55], female EFAD-Tg mice [48] were used in this study to identify the effects of Aβ pathology on *APOE* genotype-specific modulation of behavior. EFAD mice are an AD-Tg mouse model with h-apoE expressed under the regulated control of the endogenous mouse (m)-apoE promoter (*APOE*-TR) [56] and h-Aβ42 over-expressed via the 5xFAD-Tg mice, an FAD-Tg mouse model [57]. In addition, E4FAD mice were compared to 5xFAD/*APOE*-KO to address whether apoE4 imparts a loss of positive or gain of toxic function. Finally, E2FAD, E3FAD, E4FAD and 5xFAD/*APOE*-KO mice at 2-, 4-, and 6-months of age were used as previous data demonstrated significant age-dependent (2-6 months), apoE isoform-specific (apoE4 > apoE3 = apoE2) effects on the development of Aβ pathology in EFAD mice [48,58,59]. Therefore, from a translational perspective, it is important to incorporate sex, *APOE* genotype, and Aβ pathology in a preclinical model. Using the recently developed, tractable EFAD-Tg mice and 5XFAD/*APOE*-KO mice, age-dependent changes in spatial recognition memory (Y-maze and Morris water maze), pre-synaptic (synaptophysin) and post-synaptic (PSD95 and drebrin) protein levels, and the NMDAR subunits levels and activation of the CaMK-II-CREB-BDNF pathway were measured in 2-, 4- and 6-month female mice. The results demonstrate a greater age-induced deficit in behavior and reduction in postsynaptic proteins in the E4FAD and 5XFAD/*APOE*-KO mice compared with E2FAD and E3FAD mice, consistent with an apoE4 loss of function. However, further results demonstrate that while phosphorylated CaMK-II (p-CaMK-II) followed the same apoE-specific pattern as cognition and synaptic protein levels, levels of phosphorylated CREB (p-CREB) and BDNF demonstrate an apoE4 toxic gain of function.

## Results

### Age-dependent decline in E4FAD mice in Y-maze spatial recognition memory test and deficits in E4FAD and 5xFAD/*APOE*-KO mice compared to E3FAD and E2FAD mice

**Figure 1:**
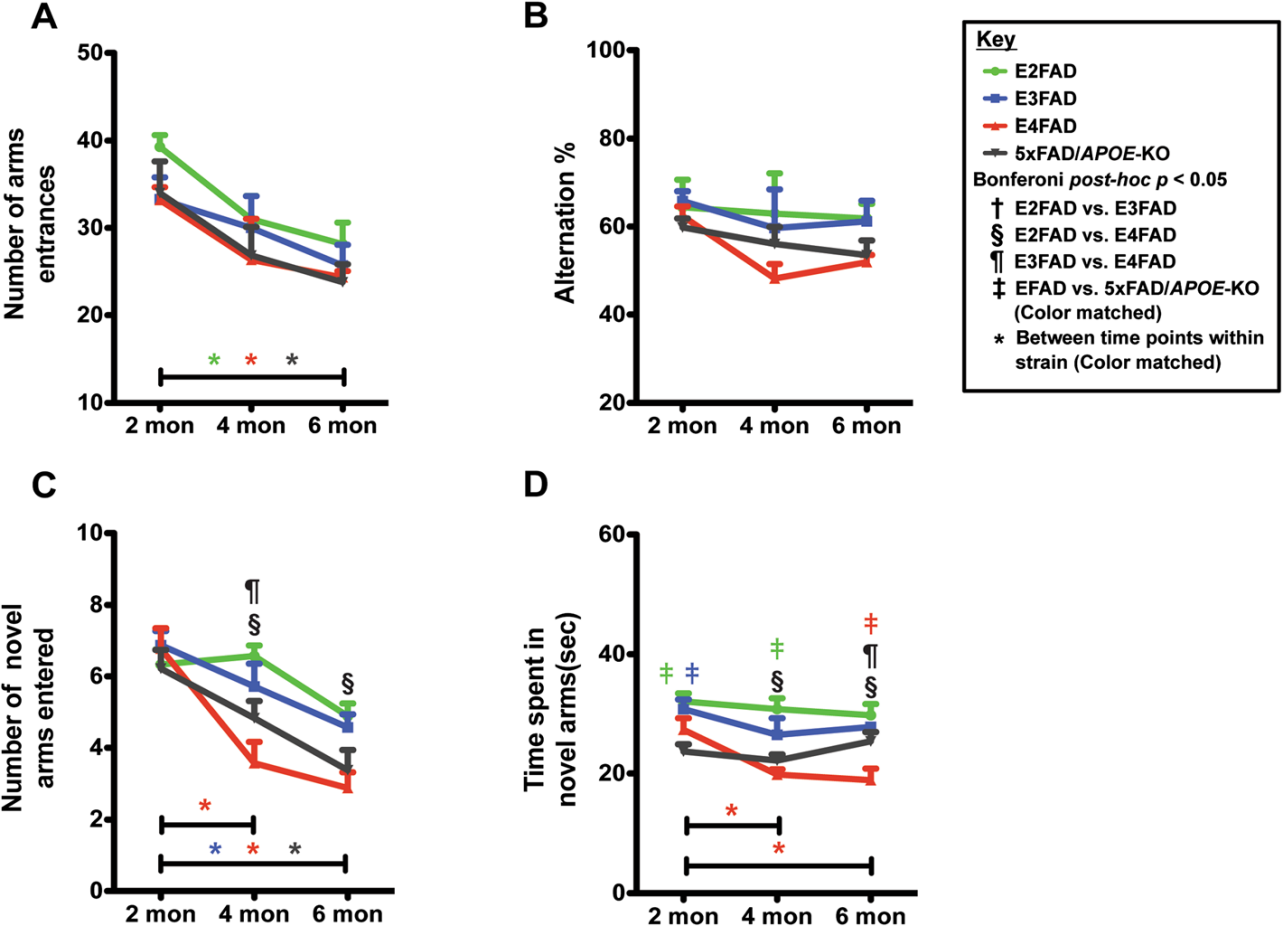
**Age-dependent decline in Y-maze of performance in E4FAD mice compared to E3FAD and E2FAD mice.** Results at 2-, 4-, and 6-months of age for E2FAD, E3FAD, E4FAD, and 5×FAD/*APOE*-KO mice: Y-maze results for **(A)** the total number of arm entries, **(B)** percent alternation, **(C)** novel arm recognition, and **(D)** time spent in novel arms. N ≥ 6 per group, expressed as means ± S.E.M. Significant differences at *p* < 0.05 via two-way ANOVA, Bonferroni *post-hoc* test identified by: † between E2FAD and E3FAD, § between E2FAD and E4FAD, ^¶^between E3FAD and E4FAD. Color matched ^‡^(green = E2FAD, blue = E3FAD, red = E4FAD, grey = 5×FAD/*APOE*-KO) between EFAD strain and 5×FAD/*APOE*-KO*.* Along the x-axis, color matched *indicates significant differences between time points within a mouse strain. There is no significant change with age unless marked.

To determine the effect of *APOE* genotype and age on cognitive function, spatial recognition memory was assessed via Y-maze in 2-, 4-, and 6-month EFAD and 5xFAD/*APOE*-KO mice. There were no significant differences in the number of arm entrances (baseline-line exploratory activity, Figure 1A) or spontaneous alternation (Figure 1B) by *APOE* genotype, however there was significance in both tests between age groups (two-way ANOVA, Additional file 1). Bonferroni *post-hoc* analysis demonstrated significantly lower arm entrances between 2 and 6 month old mice for E2FAD, E4FAD, and 5×FAD/*APOE*-KO (p > 0.05) (Figure 1A). Two-way ANOVA analysis showed a significant age effect for spontaneous alternation, however Bonferroni *post-hoc* analysis revealed no significance (Figure 1B). Thus, subsequent effects (Figure 1C,D) were not significantly influenced by differences in spontaneous exploratory spatial navigation.

Spatial recognition memory was assessed using the natural tendency of mice to preferentially explore novel over familiar spatial environments in a two-trial Y-maze test, measuring the number of novel arms entered (Figure 1C) and time spent in novel arms (Figure 1D). Two-way ANOVA demonstrated a genotype and age effect but not an age X genotype effect for both number of novel arms entered and time in novel arms (Additional file 1). Bonferroni *post-hoc* analysis revealed that a significant age effect was observed for the E4FAD mice from 2-4 months and from 2-6 months (Figure 1C,D), while E3FAD and 5xFAD/*APOE*-KO also decreased significantly form 2-6 in number of arms entered (1C). In comparisons among the genotypes at each age, E4FAD mice displayed deficits in spatial cognition (fewer novel arm entries) compared to E2FAD and E3FAD mice at 4 months, and compared to E2FAD mice at 6 months (Figure 1C), with no difference between E4FAD and 5xFAD/*APOE*-KO mice. Results for the time spent in the novel arms (Figure 1D) suggest that both E4FAD and 5xFAD/*APOE*-KO mice spent consistently less time in novel arms than E2FAD and E3FAD mice. Of interest, time in novel arms for E4FAD mice at 6 months is significantly lower than 5xFAD/*APOE*-KO mice, the only example of an apoE4 gain of toxic function for the Y-maze (Figure 1D). Together these results are consistent with E2FAD ≥ E3FAD > 5xFAD/*APOE*-KO ≥ E4FAD for spatial recognition memory as assessed by Y-maze.

### Deficits in spatial and learning and memory in the Morris water maze are greater in E4FAD and 5xFAD/*APOE*-KO mice compared to E3FAD and E2FAD mice

**Figure 2:**
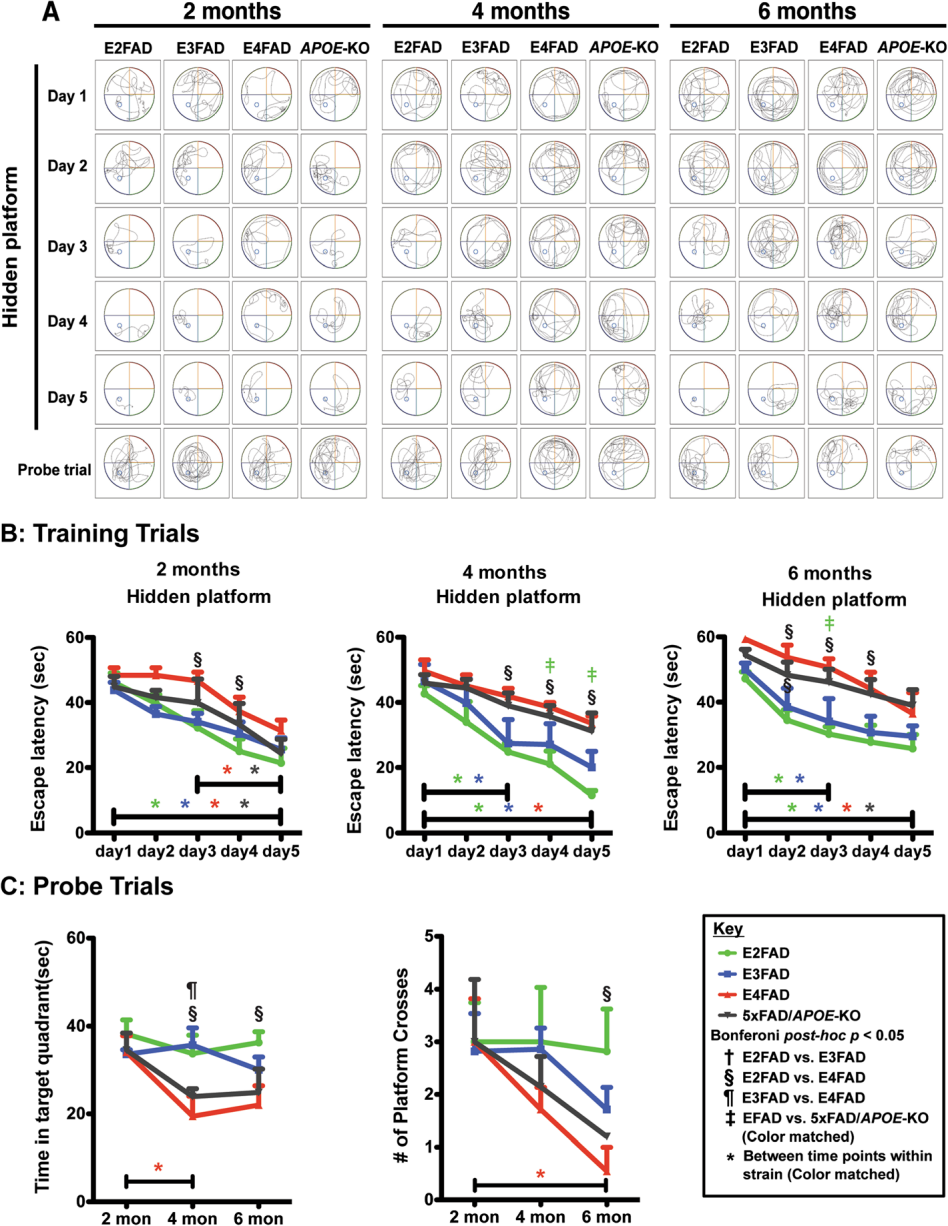
**Age-dependent decline in Morris water maze training and performance is exacerbated in E4FAD and 5xFAD/** ***APOE*** **-KO mice compared to E3FAD and E2FAD mice.** Results at 2-, 4-, and 6-months of age for E2FAD, E3FAD, E4FAD, and 5xFAD/*APOE*-KO mice: **(A)** Representative swimming tracks in Morris water maze for days 1-5 of training (*APOE*-KO = 5xFAD/*APOE*-KO). **(B)** Training trials: Escape latency for hidden platform. **(C)** Probe trials: time spent in target quadrant and number of platform crossings. N ≥ 6 per group, expressed as means ± S.E.M. Significant differences at *p* < 0.05 via two-way ANOVA, Bonferroni *post-hoc* test identified by: ^†^between E2FAD and E3FAD, § between E2FAD and E4FAD, ^¶^between E3FAD and E4FAD. Color matched ^‡^(green = E2FAD, blue = E3FAD, red = E4FAD, grey = 5xFAD/*APOE*-KO) between EFAD strain and 5xFAD/*APOE*-KO. Along the x-axis, color matched *indicates significant differences between time points within a mouse strain. There is no significant change with age unless marked.

Cognition was further assessed for spatial reference and working memory using the MWM (Figure 2). Two MWM tests were utilized to assess the capacity of mice to learn the location of a hidden platform using relevant visual cues. A 5-day training phase was used as a measure of spatial learning and memory, followed by removal of the platform for two probe trials (Figure 2C) to assess retrieval of spatial reference memory. All genotypes at each age exhibited comparable swimming speed and sensory motor functions, as determined by a visual cue test (data not shown). Thus, sensory motor or motivational effects on learning and memory performance were considered comparable. Swimming tracks for training and the probe trials were recorded for each day for each genotype and 2-, 4- and 6-month (representative example, Figure 2A).

For the 5-day training phase, the time to find the hidden platform was recorded and plotted against trial date at 2-, 4- and 6-month (Figure 2B). There was a genotype and training day effect for all age groups (two-way ANOVA, Additional file 1). Bonferroni *post-hoc* analysis revealed that for all the genotypes at each age, the time to reach the hidden platform decreased from 1 to 5 days in training phase, indicating that the mice were able to learn the task, with the exception of 5xFAD/*APOE*-KO mice at 4 months. The escape latency for the E2FAD and E3FAD mice decreased significantly from 1 to 3 days at both 4 and 6 months, while the E4FAD and 5xFAD/*APOE*-KO mice required the full 5 days for a significant learning effect at 4 and 6 months. It is also interesting to note that from 2-6 months, the escape latency, measured as the slope of the learning curve, increased from 2 to 6 months for the E4FAD (-4.26 to -2,30) and 5xFAD/*APOE*-KO mice (-5.13 to -3.35), suggesting failure of some compensatory effect over time. In comparisons among the genotypes at each age, the escape latency was longer for E4FAD compared to E2FAD mice at several training days for 2-, 4-, and 6-months (Figure 2B). This result suggests that on a given day, E4FAD mice showed delayed acquisition and poor retention of spatial information from the day before and, therefore, took longer to reach the position of the platform than the E2FAD mice. In general, the results for training trials of E4FAD were comparable to 5xFAD/*APOE*-KO mice, while E2FAD and E3FAD mice were comparable.

After 5 days of training, the platform was removed and the number of times the mice crossed the previous platform location and the time spent in the target quadrant searching for the platform were recorded (Figure 2C). There was a genotype and age effect, but not a genotype X age effect, for both probe trials (two-way ANOVA, Additional file 1). *Post-hoc* analysis by Bonferroni revealed a significant age effect for E4FAD mice for both measures, with a similar trend for the 5×FAD/*APOE*-KO. This decline is particularly dramatic for platform crosses at 6 months (Figure 2C). In comparisons among the genotypes at each age, there were no genotype effects at 2 months in either probe trial (Figure 2C). In comparisons among the genotypes at each age, the E4FAD mice spent less time in the target quadrant than both E2FAD and E3FAD mice at 4 months, and less than E2FAD at 6 months. For the number of platform crosses, the only significant difference was between E2FAD and E4FAD mice at 6 months.

The results for the MWM indicate that recently acquired spatial learning and working memory, and long-term reference memory, are impaired in E4FAD mice compared to E3FAD and E2FAD mice. These data do not support a difference between E4FAD and 5×FAD/*APOE*-KO mice (E2FAD ≥ E3FAD > E4FAD = 5×FAD/*APOE*-KO). As with Y-maze, the conclusion is that *APOE4* presents primarily as loss of function.

### Total apoE levels are lower in E4FAD mice compared to E3FAD and E2FAD mice

**Figure 3:**
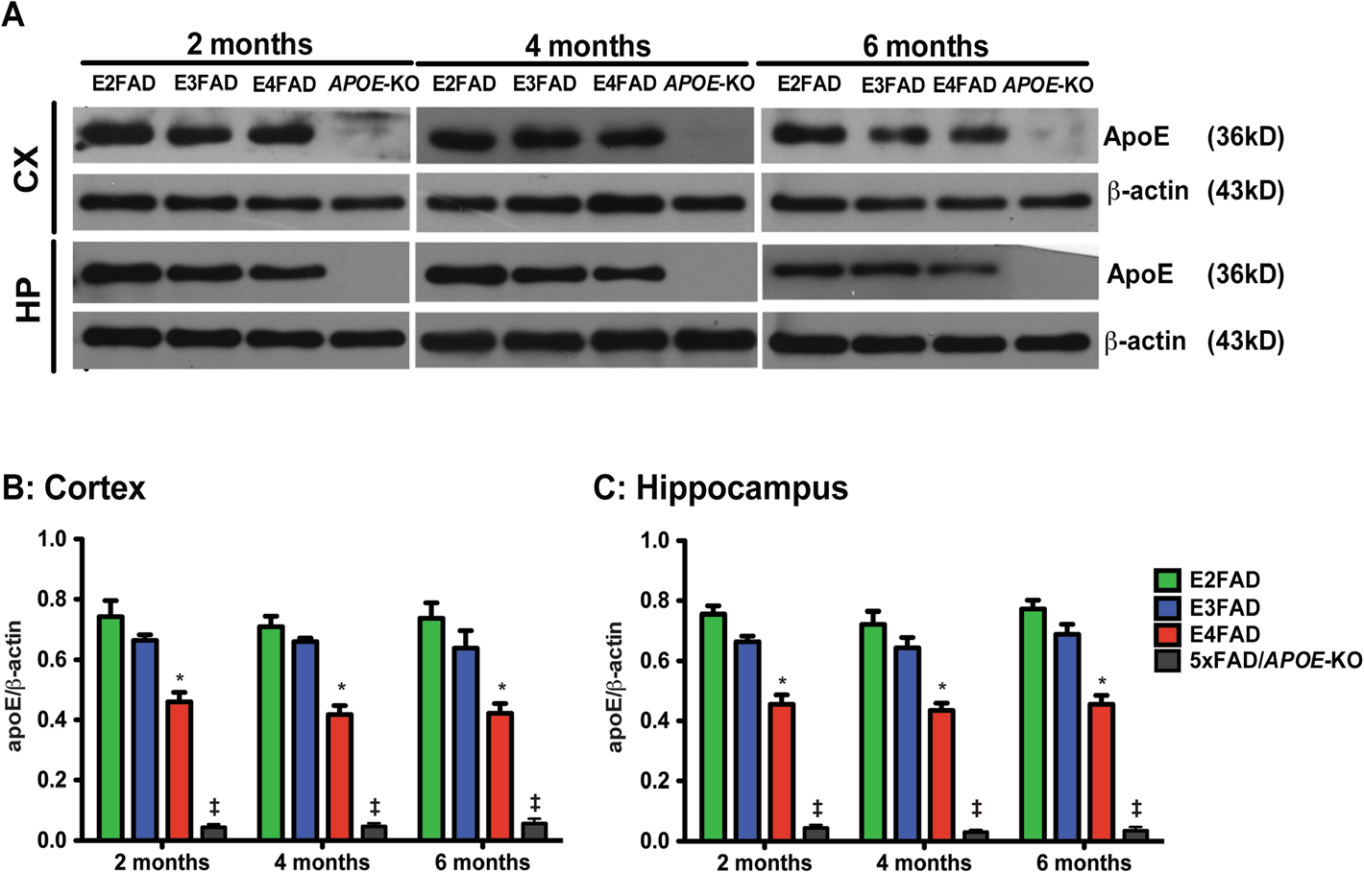
**ApoE levels are lower in E4FAD mice compared to E3FAD and E2FAD mice.** Results at 2-, 4-, and 6-months of age for E2FAD, E3FAD, E4FAD, and 5×FAD/*APOE*-KO mice: **(A)** Representative Western blot for apoE protein in cortex (CX) and hippocampus (HP) with β-actin as a control for protein loading (*APOE*-KO = 5×FAD/*APOE*-KO). Relative apoE protein levels in **(B)** CX and **(C)** HP. N = 6 per group, expressed as means ± S.E.M. Significant difference at *p* < 0.05 via two-way ANOVA, Bonferroni *post-hoc* test identified by *for E4FAD compared to E2FAD and E3FAD. No significant change between time points. ^‡^ApoE levels in 5xFAD/*APOE*-KO mice were ≥ 10-fold lower than E4FAD, *p* < 0.000001.

Total apoE (Figure 3) levels in the cortex and hippocampus of 2-, 4-, 6-month EFAD and 5xFAD/*APOE*-KO mice were measured by Western blot (representative blot Figure 3A) and normalized to β-actin. Two-way ANOVA showed only a genotype effect for apoE levels (Additional file 1). Age had no effect on apoE levels in any genotype in either brain region at any age (Figure 3B, C). However Bonferroni *post-hoc* analysis, for both brain regions at each age, total apoE4 levels were significantly lower than apoE2 and apoE3 (Figure 3B,C). These data are consistent with previous studies comparing apoE4 levels with apoE3 in humans and mice [48,60-64] and support the few published studies comparing apoE4 with apoE2 levels [48,60]. As would be expected, the levels of apoE in the 5xFAD/*APOE*-KO mice were just above level of detection and significantly lower than any of the apoE isoforms.

### Age-dependent decline in post-synaptic-related protein levels is exacerbated in E4FAD and 5xFAD/*APOE*-KO mice compared to E3FAD and E2FAD mice

**Figure 4:**
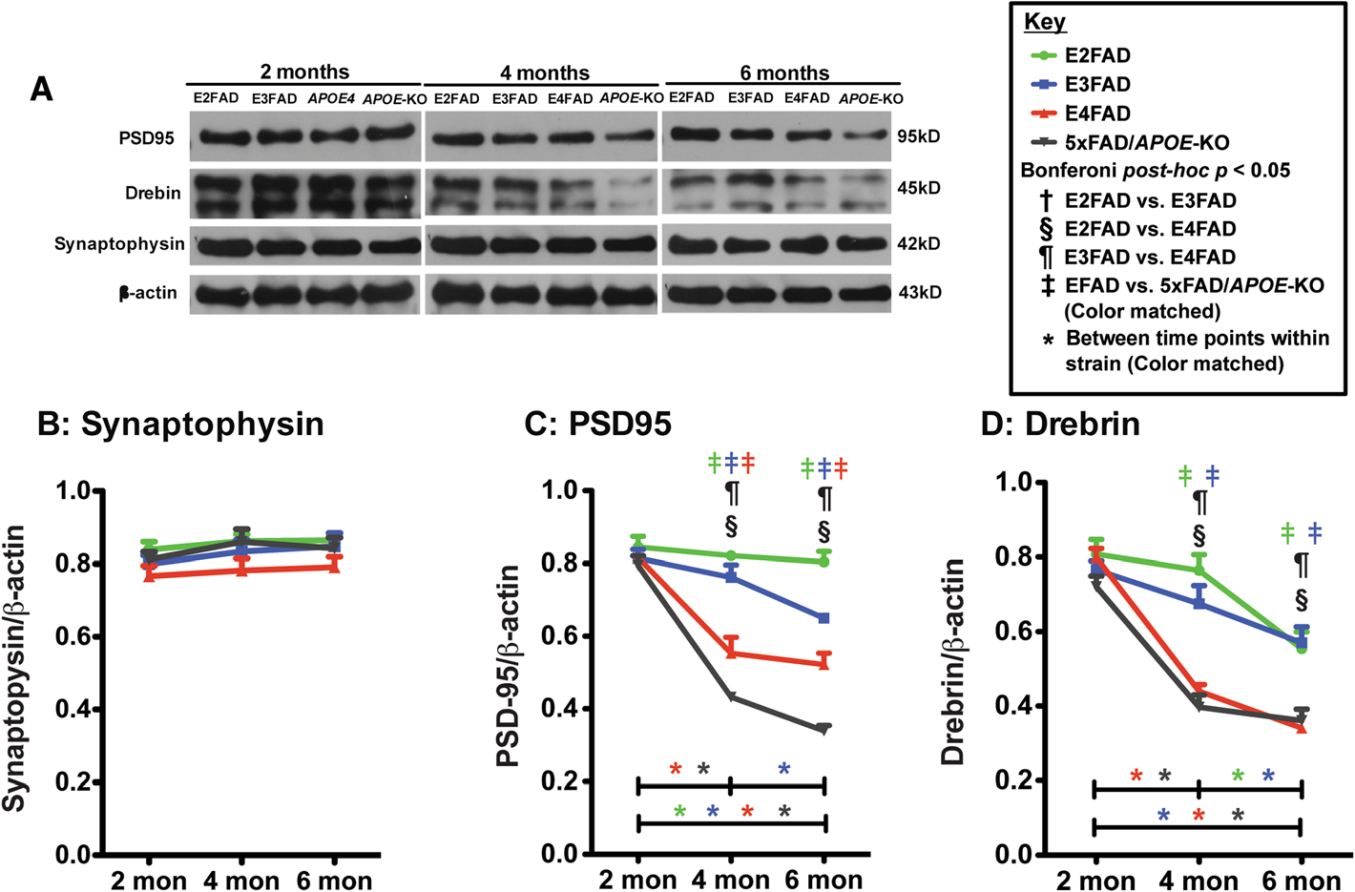
**Age-dependent decline in post-synaptic protein levels is exacerbated in 5xFAD/** ***APOE*** **-KO ≥ E4FAD mice compared to E3FAD and E2FAD mice.** Results at 2-, 4-, and 6-months of age for E2FAD, E3FAD, E4FAD, and 5xFAD/*APOE*-KO mice: **(A)** Representative Western blot images for PSD95, drebrin and synaptophysin proteins in HP with β-actin as a control for protein loading (*APOE*-KO = 5xFAD/*APOE*-KO). Relative protein levels of **(B)** synaptophysin, **(C)** PSD95 and **(D)** drebrin. N = 6 per group, expressed as means ± S.E.M. Significant differences at *p* < 0.05 via two-way ANOVA, Bonferroni post-hoc test identified by: ^†^between E2FAD and E3FAD, § between E2FAD and E4FAD, ^¶^between E3FAD and E4FAD. Color matched ^‡^(green = E2FAD, blue = E3FAD, red = E4FAD, grey = 5xFAD/*APOE*-KO) between EFAD strain and 5xFAD/*APOE*-KO. Along the X-axis, color matched *indicates significant differences between time points within a mouse strain. There is no significant change with age unless marked.

To begin to dissect potential pathways for apoE4 modulation of cognitive deficits, levels of presynaptic (synaptophysin) and postsynaptic (PSD95, drebrin) proteins were measured in the hippocampus by Western blot (representative blot Figure 4A).

There were no age or genotype effects on the levels of synaptophysin, a presynaptic protein (Figure 4B; two-way ANOVA, Additional file 1). Two-way ANOVA of PSD95 (4C) and drebrin (4C) revealed a significant effect for genotype, age and genotype X age (Additional file 1). Although post-synaptic proteins PSD95 and drebrin levels were equal among genotypes at 2 months, Bonferroni *post-hoc* analysis showed significant age effects for both proteins in all genotypes from 2-6 months with the exception of drebrin levels in the E2FAD mice. It is also interesting to note that the decrease in both PSD95 and drebrin for E4FAD and 5xFAD/*APOE*-KO were significant from 2-4 months, while E3FAD decreased significantly from 4-6 months, and PSD95 levels in E2FAD mice decreased minimally and only from 2-6 months.

In comparisons among the genotypes at each age, both PSD95 and drebrin levels in E2FAD mice were significantly higher than E4FAD and 5xFAD/*APOE*-KO at 4 and 6 months. Comparisons among genotypes demonstrate that at 4 and 6 months, PSD95 levels were E2FAD = E3FAD > E4FAD > 5xFAD/*APOE*-KO, evidence for apoE as a loss of function, although there was no difference in drebrin levels between E4FAD and 5xFAD/*APOE*-KO mice, and these drebrin levels were significantly lower than the drebrin levels in E2FAD and E3FAD mice, with the resulting summary for drebrin: E2FAD = E3FAD > E4FAD = 5xFAD/*APOE*-KO.

Collectively these data support the observation that postsynaptic proteins are affected prior to presynaptic proteins [38,39,65-67] and this effect may underlie apoE-modulated cognitive deficits. Further, as with cognitive dysfunction, apoE represents primarily a loss of positive function.

### Age-dependent decline in NMDAR subunits levels is exacerbated in E4FAD and 5xFAD/*APOE*-KO mice compared to E3FAD and E2FAD mice

**Figure 5:**
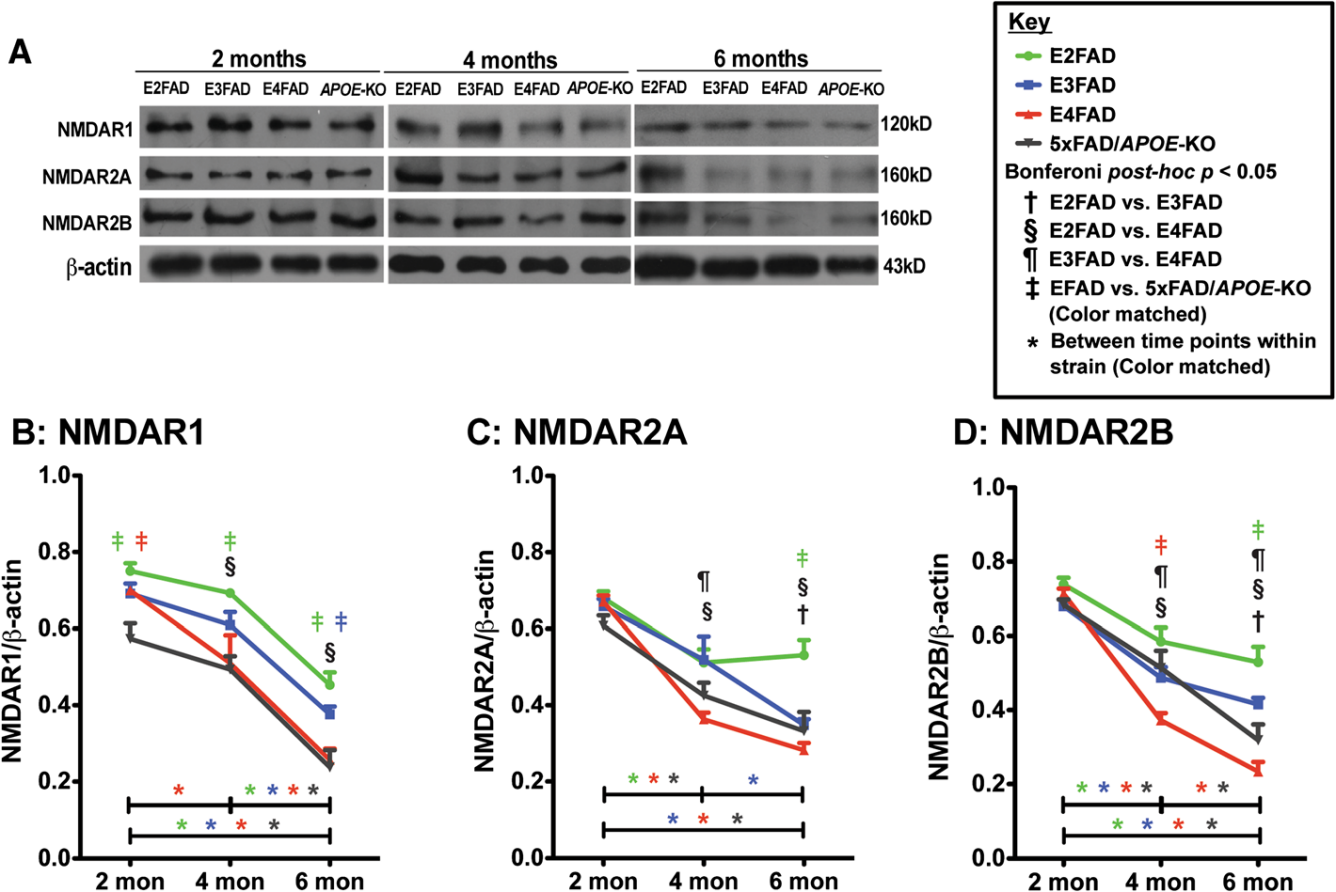
**Age-dependent decline in NMDAR subunit protein levels are exacerbated in E4FAD and 5xFAD/** ***APOE*** **-KO compared to E3FAD and E2FAD mice.** Results at 2-, 4-, and 6-months of age for E2FAD, E3FAD, E4FAD, and 5xFAD/*APOE*-KO mice: **(A)** Representative Western blot for NMDAR1, NMDAR2A and NMDAR2B proteins in HP with β-actin as a control for protein loading (*APOE*-KO = 5xFAD/*APOE*-KO). Relative protein levels of **(B)** NMDAR1, **(C)** NMDAR2A, and **(D)** NMDAR2B. N = 6 per group, expressed as means ± S.E.M. Significant differences at *p* < 0.05 via two-way ANOVA, Bonferroni post-hoc test identified by: ^†^between E2FAD and E3FAD, § between E2FAD and E4FAD, ^¶^between E3FAD and E4FAD. Color matched ^‡^(green = E2FAD, blue = E3FAD, red = E4FAD, grey = 5xFAD/*APOE*-KO) between EFAD strain and 5xFAD/*APOE*-KO. Along the x-axis, color matched *indicates significant differences between time points within a mouse strain. There is no significant change with age unless marked.

Evidence indicates that reduced postsynaptic NMDAR levels are involved in cognitive dysfunction in AD [37-39]. Therefore*,* levels of the NMDAR subunits NMDAR1 (Figure 5B), NMDAR2A (Figure 5C) and NMDAR2B (Figure 5D) were measured in the hippocampus by Western blot (representative blot Figure 5A).

Two-way ANOVA of NMDAR results show a significant genotype and age, but no genotype X age effect (Additional file 1). Further Bonferroni *post-hoc* analysis revealed that at 2 months, the three NMDAR subunits levels were equal among genotypes except for lower levels of NMDAR1 in 5xFAD/*APOE*-KO mice, indicating a loss of apoE4 positive function compared to apoE-KO. After 2 months, all three NMDAR subunits in all genotypes decreased from 2-6 months (Figure 5B,C,D), with the exception of, again, E2FAD, consistent with the results for NMDAR2A (Figure 4C). Comparisons among genotypes demonstrate the NMDAR1 levels are consistently higher in E2FAD mice compared to the other genotypes, a trend is also observed for the levels of NMDAR2A and NMDAR2B. While the general trend for the NMDAR subunits is E2FAD and E3FAD being higher than E4FAD and 5xFAD/*APOE*-KO, it is significant to note that NMDAR2B levels are significantly lower in E4FAD compared to 5xFAD/*APOE*-KO mice at 4 months, with the trend continuing to 6 months, an example of apoE4 gain of toxic function (Figure 5D).

Overall, consistent with cognition and levels of postsynaptic proteins, these data indicates that apoE mediates primarily a loss of positive function with NDMAR subunits levels: E2FAD ≥ E3FAD > E4FAD ≈ 5xFAD/*APOE*-KO.

### Age dependent decline in p-CaMK-II levels is significant in E3FAD, E4FAD and 5xFAD/*APOE*-KO mice compared to E2FAD mice; age dependent decline in p-CREB and BDNF is exacerbated in E4FAD > 5xFAD/*APOE*-KO ≥ E3FAD > E2FAD

**Figure 6:**
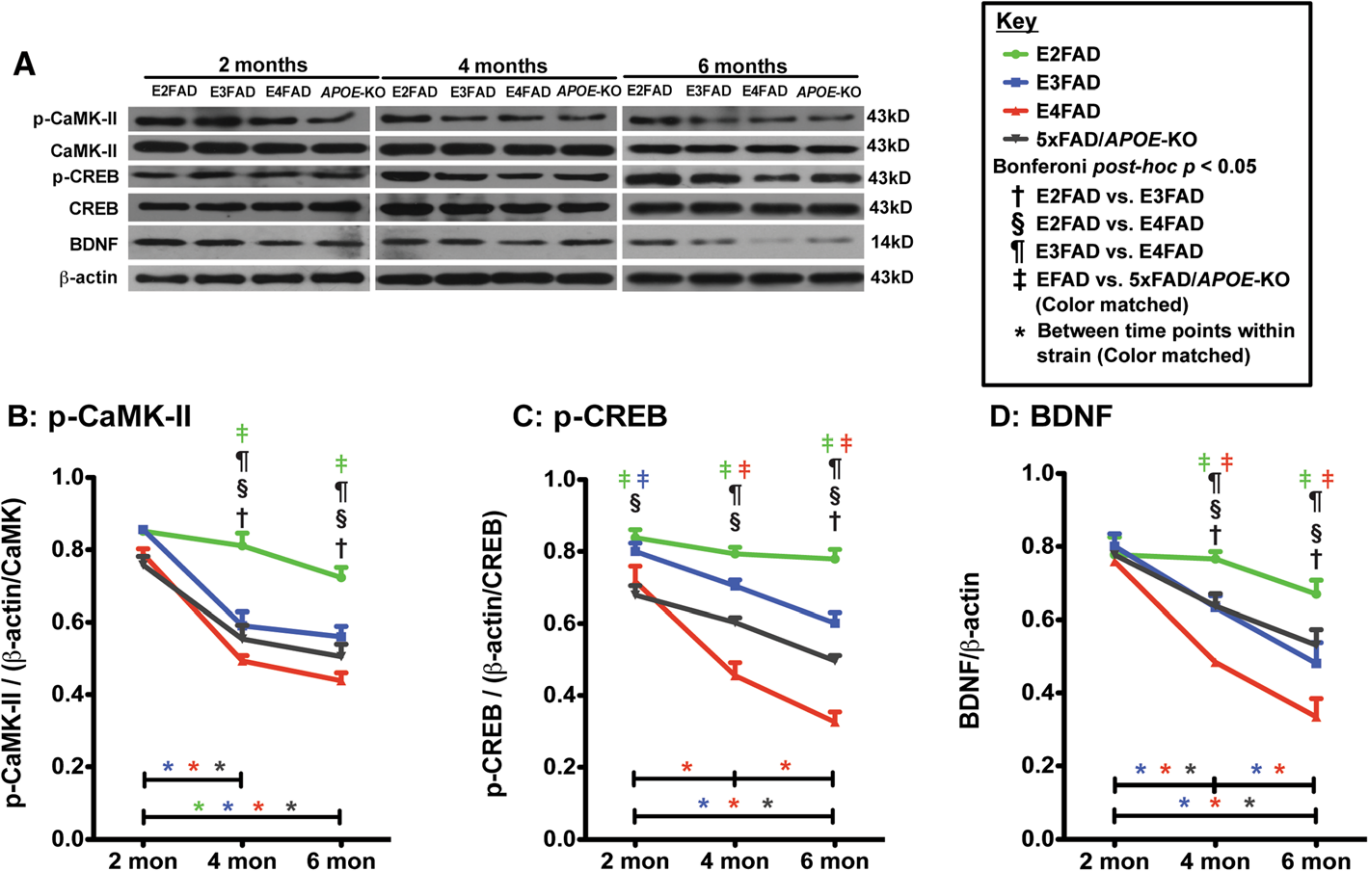
**Age-dependent decline in NMDAR-mediated signaling proteins is exacerbated in E4FAD ≥ 5xFAD/** ***APOE*** **-KO ≥ E3FAD > E2FAD mice.** Results at 2-, 4-, and 6-months of age for E2FAD, E3FAD, E4FAD, and 5xFAD/*APOE*-KO mice: **(A)** Representative Western blot images for p-CaMK-II, p-CREB, and BDNF proteins in HP with β-actin/CREB, β-actin/CaMK-II or β-actin as a control for protein loading, respectively (*APOE*-KO = 5xFAD/*APOE*-KO). Relative protein levels of **(B)** p-CaMK-II, **(C)** p-CREB and **(D)** BDNF. N = 6 per group, expressed as means ± S.E.M. Significant differences at *p* < 0.05 via two-way ANOVA, Bonferroni *post-hoc* test identified by: ^†^between E2FAD and E3FAD, ^§^between E2FAD and E4FAD, ^¶^between E3FAD and E4FAD. Color matched ^‡^(green = E2FAD, blue = E3FAD, red = E4FAD, grey = 5xFAD/*APOE*-KO) between EFAD strain and 5xFAD/*APOE*-KO. Along the x-axis, color matched *indicates significant differences between time points within a mouse strain. There is no significant change with age unless marked.

The NDMAR subunit levels were significantly lower in E4FAD, with a trend in 5xFAD/*APOE*-KO mice, compared to E2FAD and E3FAD mice. As reduced levels/activation of the NMDAR pathway CaMK-II/CREB/BDNF [42,68,69] are observed in AD patients and associated with impaired neuronal function *in vitro* and *in vivo* [70-73], these downstream signaling molecules, specifically the levels of BDNF (Figure 6D) and activated p-CaMK-II (Figure 6B) and pCREB (Figure 6C), were measured in the hippocampus by Western blot (representative blot Figure 6A). Two-way ANOVA on the p-CaMK-II, p-CREB, and BDNF results showed a significant effect genotype, age and genotype X age interaction (Additional file 1), Bonferroni *post-hoc* analysis are detailed below.

*p-CaMK-II.* No differences in p-CaMK-II levels were observed among the genotypes at 2 months (Figure 6B). After 2 months, p-CaMK-II levels decreased from 2-6 months in all the genotypes, although the decrease from 4-6 months was not significant in only E2FAD mice. Comparison among the genotypes at 4 and 6 months, revealed p-CaMK-II levels: E2FAD > E3FAD = E4FAD = 5xFAD/*APOE*-KO.

*p-CREB.* At 2 months, p-CREB levels were higher in E2FAD and E3FAD mice compared to 5xFAD/*APOE*-KO mice, and E4FAD levels were lower E2FAD mice (Figure 6C). From 2-6 months, p-CREB levels decreased significantly in all the genotypes but E2FAD. Comparison among the genotypes revealed at 6 months, with a similar trend at 4 months, the levels of p-CREB for the genotypes was E2FAD > E3FAD > 5xFAD/*APOE*-KO > E4FAD, consistent with a gain of toxic function for apoE4.

*BDNF.* As observed for p-CaMK-II, BDNF levels were not different among the genotypes at 2 months Figure 6D). After 2 months, BDNF levels decreased with age in the E3FAD, E4FAD and 5xFAD/*APOE*-KO mice, while levels in E2FAD mice did not change. Comparison among the genotypes revealed that at both 4 and 6 months, the levels of BDNF for the genotypes was E2FAD > E3FAD ≥ 5xFAD/*APOE*-KO > E4FAD. As with p-CREB, levels of BDNF are consistent with a toxic gain of function for apoE4*.*

Thus, in contrast to cognitive dysfunction, postsynaptic protein levels (PSD95, drebrin, NDMAR), and p-CaMK-II where apoE4 appears to be a loss of function compared to apoE-KO*,* apoE4 demonstrates a toxic gain of function with p-CREB and BDNF levels.

## Discussion

*APOE4*-induced AD risk is likely the result of multiple, overlapping mechanisms, both Aβ-dependent and Aβ independent (for review [74]). One challenge in understanding the effect of *APOE* genotype on various mechanistic readouts is determining whether apoE4 represents a loss of positive function or a gain of toxic function. Thus, we investigated the early, age-dependent *APOE* genotype-specific effects on cognitive functions and synaptic viability in EFAD-Tg mice [48,58,59], specifically female mice based on data in both humans [53-55] and Tg mice [33,35,36,75,76] that *APOE4* females exhibit significantly increased cognitive impairment compared to *APOE4* males and *APOE3* females. In the Y-maze, a significant age-dependent decline in spatial recognition memory was observed only for the E4FAD mice from 2-4 months, indicating a more rapid decline at earlier stages of Aβ deposition compared to other genotypes (Figure 1). In the MWM, a measure of spatial learning and memory, the E4FAD and 5xFAD/*APOE*-KO mice were both slower to learn than the E2FAD and E3FAD mice during the 5-day training phase (Figure 2B). In addition, both the E4FAD and 5xFAD/*APOE*-KO mice exhibited an age-related increase in escape latency from 2-6 months during the training trials, suggesting the failure of some compensatory effect over time. As this is consistent with previous studies demonstrate higher anxiety levels in *APOE4-TR* and *APOE*-KO mice [76,77], we hypothesize that this elevated stress response may facilitate spatial learning in young E4FAD mice and mask adverse effects of apoE4 on spatial cognition. Indeed, it has been shown that normal aging can counteract stress-induced facilitation of cognitive processing in *APOE4*-TR mice, as measured by MWM, making phenotypic differences easier to detect in older mice [33,75]. This apoE4 effect in the EFAD mice is amplified by the overproduction of Aβ42 driven by the presence of the 5-FAD mutations. Indeed, the 5xFAD mice show progressive learning and memory deficits tasks as early as 3 months [78-82]. As deficits in spatial learning and memory due to apoE4 have mainly been reported in older and non-AD mice [33,75], our findings are consistent with synergistic effects between apoE4 and the aggressive Aβ42 pathology characteristic of the EFAD mice [48]. In addition, the use of only female EFAD mice also optimized the risk of cognitive deficits in the E4FAD mice. Indeed, sex interacts with *APOE* to affect cognitive function. Clinical data indicate that the *APOE4*-induced risk for AD is significantly greater, perhaps exclusive to, females [53-55]. These data are consistent with the greater cognitive impairment in female *APOE4*-TR mice compared to female *APOE3*-TR mice, and with both *APOE3*- and *APOE4*-TR females compared to *APOE* genotype-matched males (review [35]). Overall, as measured in this study, behavior appeared to be primarily an apoE4 loss of function, specifically: E2FAD = E3FAD > E4FAD ≈ 5xFAD*/APOE*-KO. However, further studies in humans and Tg-mouse models are critical to determine the role of potential interactive effects among Aβ pathology, *APOE* genotype and sex on memory and cognitive decline.

**Figure 7:**
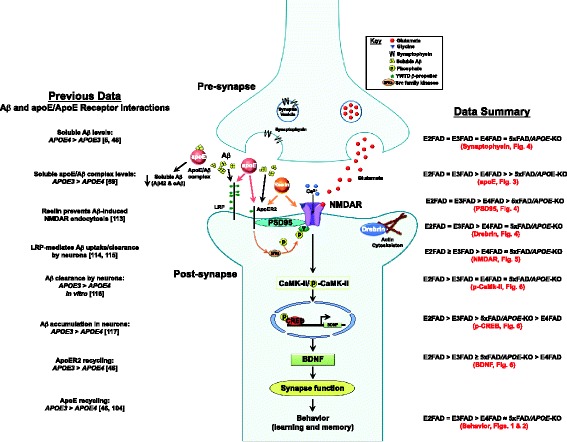
**Diagram of potential** ***APOE4*** **-induced decrease in synapse-related proteins and disruption of NMDAR-mediated signaling, resulting in impaired learning and memory.** With *APOE4*, the levels of key post-synaptic proteins are reduced, including the subunits of NMDAR. NMDAR signaling via activation by phosphorylation of CaMK-II and CREB to increase expression of BDNF is also disrupted by *APOE4*, compromising synaptic function and impairing learning and memory. Previous work has demonstrated that apoE isoform-specific effects on Aβ clearance and interactions with apoE receptors likely play a role in this process at several levels. Soluble levels of Aβ are lower with apoE3 and inversely correlated with levels of apoE3/Aβ complex [59], suggesting a potential clearance mechanism. LRP mediates Aβ uptake by neurons [101,102], and, *in vitro*, Aβ clearance is impaired with apoE4 [103], consistent with a greater accumulation of intraneuronal Aβ [104] compared to apoE3. For ApoER2, ligand recycling is impaired with apoE4 compared to apoE3 [46]. Loss of ApoER2 reduces Reelin binding, thus reducing activation of NMDAR via signaling by the Src family kinases [46,105], leading to decreased synaptic function and therefore decreased learning and memory.

Spatial and learning memory performances are directly linked to synaptic function. ApoE4 is associated with progressive synaptic deficits in both AD patients and h-*APOE-*Tg mouse models [83-87]. Consistent with previous reports, age-induced reductions in synaptic proteins preferentially occurred in post-synaptic proteins compared particularly to synaptophysin, a pre-synaptic protein (Figure 4) [81,88-90]. In the current model, levels of drebrin and PSD95 were lower in E4FAD and 5xFAD*/APOE*-KO mice compared to E2FAD and E3FAD mice, consistent with an apoE4 loss of function. ApoE is also linked to long-term potentiation (LTP) and NMDAR-mediated signaling [50,51]. As the NMDAR component of synaptic transmission has been shown to decline during aging [91,92], NMDAR activation may provide a mechanistic pathway for understanding apoE-related memory impairment. Indeed, in this study apoE-related cognitive impairment correlates with a decrease in the levels of NMDR subunits and components of the signaling pathway (p-CaMK-II/p-CREB/BDNF). Levels of all three NMDAR subunits were reduced with age for all the genotypes with reductions greater in the E4FAD and 5xFAD*/APOE*-KO mice compared to E2FAD and E3FAD mice, consistent with an apoE4 loss of function (Figure 5). These apoE4-related deficits in NMDAR-dependent functions likely reflect changes to neuronal networks contributing to short and long-term memory, and their contribution to memory consolidation [93-96]. Reduction of BDNF levels through either genetic or pharmacological means not only impaired LTP and reduced the number of synapses, but also caused deficits in the formation and consolidation of memory [97-99]. However, the effects of *APOE* genotype on the p-CaMK-II, p-CREB, and BDNF signaling cascade are not consistent. Again, all the signaling components were reduced with age for all the genotypes (Figure 6). While the greater reduction in p-CaMK-II in E4FAD and 5xFAD/*APOE*-KO mice compared to E2FAD and E3FAD mice is consistent with an *APOE4* loss of function*,* apoE4 represents a gain of toxic function for both the activated p-CREB transcription factor and its downstream target protein BDNF, as the reductions in p-CREB and BDNF levels are greater in E4FAD compared to 5xFAD/*APOE*-KO mice (summarized in Figure 7, right side). This gain of toxic function does not translate directly to the behavioral measures used in this study. One likely possibility for this seeming disconnect is that a number of signaling pathways are activated by changes in synaptic proteins, particularly the NMDAR, that eventually converge to modulate behavioral readouts. Thus, the final effect on behavior will be a composite of a number of signaling pathways (reviewed in [100]). Taken together, our findings support the notion that apoE4 exacerbates behavioral deficits in EFAD mice by decreasing synapse viability by reducing synapse-related proteins, particularly via down-regulation of NMDAR and NMDAR-mediated signaling via CaMK-II, CREB, and BDNF (Figure 7). Consistent with the *APOE2* protective effect for AD risk [1-11], these results also demonstrate that E2FAD mice are consistently less susceptible to age-induced changes in the components of this cascade, from signaling to behavior.

ApoE is the primary ligand for the low-density lipoprotein (LDL) receptor (LDLR) family (apoE receptors), although Reelin is the primary ligand for ApoE-receptor 2 (ApoER2). ApoER2 and Reelin are important modulators of synaptic plasticity and NMDAR functions *in vitro* and *in vivo* [106-108]. Thus, the association between ApoER2, Reelin and NMDAR are critical for LTP, memory formation and retrieval. ApoE4 has been demonstrated to reduce the cell-surface levels of both ApoER2 and NMDAR via intracellular sequestration, thus inhibiting the ability of Reelin to facilitate glutamate-mediated synaptic plasticity [46]. The impaired recycling of apoE4 may contribute to this reduction in receptors at the cell surface [105]. Loss of ApoER2 reduces Reelin binding, thus further reducing activation of NMDAR via signaling by the Src family kinases [46,105].

While a number of Aβ-independent mechanisms likely contribute to the *APOE*-associated risk for AD [109], oAβ has been demonstrated to be preferentially synapotoxic (for review [37,110]). We have published the effects of *APOE* genotype on Aβ accumulation in the EFAD mice at 6-months of age, the age of the mice used for this study [48,58,59,111,112]. These results demonstrate amyloid deposition by IHC and total brain Aβ42 by ELISA is: 5xFAD > E4FAD > E3FAD = E2FAD. A three-step sequential protein extraction protocol using TBS (soluble), TBS + Triton X-100 (TBSX, detergent), and formic acid (FA, insoluble) was used for the hippocampus and cortex. In the soluble fractions of both brain regions, both Aβ42 and oAβ are: E4FAD > E3FAD = E2FAD. There is no *APOE* genotype difference in the levels of Aβ42 in the detergent fraction. In the insoluble fraction, Aβ42 is: E4FAD > E3FAD = E2FAD. As the EFAD mice are on the 5xFAD background, the amount of Aβ40 is difficult to detect; the primary species is Aβ42. Thus, Aβ levels (amyloid, soluble and insoluble) are greatest in the E4FAD mice. This association between *APOE* and Aβ accumulation is consistent with the functional changes reported herein. Therefore, a particularly relevant approach to interpreting the results of this study is to consider *APOE* modulation of soluble Aβ levels at the synapse (Figure 7, left side). Previous publications from our group and others demonstrate that apoE isoform-specific effects on Aβ clearance and interactions with apoE receptors likely play a role in this process at several levels. It has been specifically demonstrated that apoE4 both increases the levels of oAβ and directs it to the synapse [5]. ApoE isoforms may modulate oAβ levels through differential apoE/Aβ complex levels [113]. However, as isolation and analysis of the apoE/Aβ complex *in vivo* is technically challenging, data are conflicting as to the significance or even the existence of this complex [114-116]. Nevertheless, it is interesting to note that the levels of soluble apoE4/Aβ complex are lower than apoE3/Aβ and decrease in AD in human synaptosomes, CSF and EFAD-Tg mouse brains, the reverse of soluble oAβ levels [48,58,59,113]. ApoE receptors also play a key role, particularly ApoER2, as Reelin signaling can prevent the oAβ-induced inhibition of NMDAR at the synapse [117]. As well, neuronal LRP1 provides a significant mechanism for the clearance Aβ [101,102] and *in vitro*, Aβ clearance is impaired with apoE4, compared to apoE3 [103], consistent with a greater accumulation of intraneuronal Aβ [104]. To connect these Aβ-dependent processes to decreased synaptic function and impaired cognition requires, in part, a return to ApoER2, NMDAR and Reelin. ApoE4 induces deficits in ApoER2 and NMDAR signaling and recycling, as well as reducing Reelin binding to both receptors [46,105]. Ultimately, these apoE-mediated differential effects on apoE receptors and Aβ accumulation could contribute to the mechanisms responsible for synaptic dysfunction and cognitive decline characteristic of observed in the EFAD mice that, ultimately, will translate to AD patients.

## Conclusions

Herein we provide evidence that the *APOE4* genotype constitutes a loss of positive function contributing to age-related deficits in behavioral performances in the EFAD and 5xFAD/*APOE*-KO mice (Figure 7-right side; Table 1A). This loss of positive function with apoE4 was related to a decrease in post-synaptic proteins, including NMDAR subunits, leading to impaired NMDAR-related signaling. However, apoE4 represents a gain of toxic function for the final components measured in this pathway, activated p-CREB and its downstream target protein BDNF. It is our interpretation that multiple signaling pathways converge to determine the final synaptic transmission impairment and learning and memory deficits associated with apoE4. This conclusion requires further study to determine the potential contributions of other signaling components to either an apoE4-mediated loss or gain of function, data of high therapeutic significance. Table 1B provides a representative summary of CNS-relevant functions modulated by apoE4 that can be attributed to a loss of positive or gain of toxic function. Again, it is important to assess whether apoE4 imparts a loss of positive or gain of toxic function in comparison to the absence of apoE (*APOE*-KO), not simply a comparison to apoE2/apoE3. Table 1B provides a larger perspective on the interplay between among multiple functions that exhibit a loss of positive (for example, anti-inflammatory properties and amyloid deposition) or gain of toxic function (for example, toxic proteolytic fragments of apoE and oAβ-induced neurotoxicity *in vitro* and *ex vivo*). Only an understanding of the relative contribution of the functions measured in this paper, listed in Table 1A and the many others as yet undefined will enable a confident identification of *APOE4*-induced AD risk as a loss of positive or gain of toxic function.

**Table 1:** **Evidence that** ***APOE4*** **is a loss of positive or gain of negative function: comparison to absence of** ***APOE***

**A. Within manuscript**			
**Measure**	**apoE4 represents:**	**Mouse model**	
Behavior/cognition	Loss of function	5xFAD/*APOE*-KO, EFAD	
Postsynaptic protein levels	Loss of function		
NMDAR subunits	Loss of function		
p-CaMK-II levels	Loss of function		
p-CREB levels	Gain of toxic function		
BDNF levels	Gain of toxic function		
**B. Literature overview**			
**Measure**	**apoE4 represents:**	**Mouse model**	**Reference**
Anti-inflammatory	Loss of function	*APOE*-KO, *APOE*-TR	[47]
Baseline LTP	Loss of function	WT, *APOE*-KO, *APOE*-TR *(Ex vivo* hippocampal slice cultures)	[50]
Amyloid deposition	Loss of function	*APOE*-KO x APP^V717+/-^	[118],
		APP^V717+/-^ x GFAP-apoE^+/-^	[119,120],
		5xFAD, EFAD	[48]
			for review, [35]
ApoE lipidation	Loss of function	*APOE*-TR	[121]
		EFAD	[58]
Dendritic spine density	Loss of function	WT, *APOE*-KO, GFAP-apoE^+/+^	[84]
BBB integrity	Loss of function	WT, *APOE*-KO, *APOE*-TR, GFAP-*APOE*	[122]
Aβ clearance across the BBB	Loss of function	WT, APOE^+/-^, APOE-KO	[123]
Behavior/cognition	Gain of toxic function	WT, *APOE*-KO, *APOE*-TR	[33]
		WT, *APOE*-KO, *APOE*-TR	[34]
		WT, *APOE*-KO, GFAP-apoE	[124]
		WT, *APOE*-KO, GFAP-apoE (female)	[125]
		WT, *APOE*-KO, NSE-apoE	[124,126]
Accumulation of intraneuronal oAβ	Gain of toxic function	*APOE*-KO, *APOE*-TR	[127]
oAβ-induced neurotoxicity	Gain of toxic function	WT, *APOE*-KO, *APOE*-TR	[52]
		*In vitro* neuron/glial co-cultures	
oAβ-dependent inhibition of LTP	Gain of toxic function	*APOE*-KO, *APOE*-TR	[51]
Neurotoxicity of apoE proteolytic fragments	Gain of toxic function	Variety with *APOE*-KO control	[75,128] for review, [129]
Neurite outgrowth	Loss of function	*APOE*-KO olfactory epithelia cultures (exogenous apoE added) *APOE*-KO	[130]
	Gain of toxic function	cortical neuron cultures (exogenous apoE added)	[131]

Targeting the most potent genetic risk factor for AD appears a very attractive strategy and is still under intense study. If the hypothesis is that all apoE isoforms, particularly apoE4, represent a toxic gain of function, then reducing *APOE* expression and/or apoE levels is one therapeutic approach for AD. However, the potential dangers of this approach in the human brain are still subjected to debate [30]. Here, we provide additional insight into the mechanism by which *APOE4* increases AD risk, in which apoE4 mainly appears as a loss of positive function. Accordingly, rather than *APOE* gene inactivation, therapies that correct the loss of positive function related to apoE4, such as increasing the lipidation of apoE4 containing lipoproteins [58] appear to be more appropriate.

## Methods

### Animals

All experiments were conducted in accordance with the rules and regulations of the Institutional Animal Care and Use Committee protocols at Fujian Medical University, in conformance with international guidelines for the ethical use of animals. Investigators conducting the sample processing and analyses were blinded for *APOE* genotype and age. The 5xFAD/*APOE*-KO and EFAD mice (E2FAD, E3FAD, and E4FAD) were supplied by the LaDu lab. The EFAD mice [48] were originally generated by crossing 5xFAD mice [57] and h-*APOE*-TR mice [56]. 5xFAD mice express APP K670N/M671L + I716V + V717I and PS1 M146L + L286V under the control of the neuron-specific mouse Thy-1 promoter, resulting in the overproduction of specifically Aβ42 [57]. In *APOE*-TR mice, the coding domain of m-apoE is replaced by h-apoE2, apoE3 or apoE4 [56]. Thus, EFAD mice are *APOE*-TR^+/+^/5xFAD^+/-^ [48]. The 5xFAD/*APOE*-KO mice were made by knocking-out m-*APOE* from the 5xFAD mice.

### Behavioral tests

Spatial/reference memory was assessed in EFAD mice first using the Y-maze test, followed by the Morris water maze (MWM) test, as previous described [90,132]. *Y-maze*. Spontaneous alteration including total activity and percentage spontaneous alternation/exploration was initially determined as a measure of normal spatial navigation. Short-term spatial recognition memory was then assessed using a two-trial protocol with 10 minute (min) training (trial 1), 4 hour (hr) inter-trial interval and a 5 min retention trial (trial 2) for number of entries and time spent in each arm. *MWM.* Acquisition trials (training) consisted of 4 trials (maximum 1 min) a day for 5 consecutive days with escape latency recorded for each trial. Reference memory was assessed on the sixth day in a one trial test for time spent in the target quadrant and the number of times the original area of the platform was crossed.

### Tissue harvest and western blotting

2-, 4- and 6-month EFAD mice were anesthetized with sodium pentobarbital (50 mg/kg), transcardially perfused with ice-cold PBS, brains removed and dissected into cortex and hippocampus, snap-frozen in liquid nitrogen and stored at -80°C, as previous described [133]. Dissected brains were homogenized in lysis buffer [90,132] (50 mM Tris-HCl, 150 mM NaCl, pH7.4, 1% Triton X-100, 1x protease inhibitor cocktail) and 40 μg of total protein (BCA protein assay kit; Pierce, Rockford, IL) was separated on 4–12% gradient Bis-Tris gels (Invitrogen) under reducing conditions, and transferred to PVDF membranes [47]. The following primary antibodies were used: rabbit anti-PSD95 (1:3000, Abcam), mouse anti-synaptophysin (1:2000, Abcam), mouse/rabbit anti-β-actin (1:2000; Abcam), rabbit anti-drebrin antibody (1:1000; Abcam), rabbit anti-NMDAR1/anti-NMDAR2B (1:1000; Millipore), anti-NMDAR2A (1:500; Millipore), mouse anti-apoE (1:600; Santa Cruz), rabbit anti-BDNF (1:200; Santa Cruz), rabbit anti-p-CaMK-II (1:1000; Santa Cruz) and rabbit anti-p-CREB (1:1000; Cell Signaling) [90,132]. HRP-conjugated secondary antibodies, enhanced chemiluminescence (Amersham, Piscataway, NJ) and Image J software were used to quantify densities of the immunoreactive bands relative to β-actin.

### Statistical analysis

Data are expressed as mean ± standard error mean (S.E.M.). Data were analyzed by two-way analysis of variance (ANOVA), followed by Bonferroni *post-hoc* using GraphPad Prism version 4 for Macintosh. The 2-way ANOVA tables for each Figure have been added as Additional file 1. Differences for age and genotype were considered significant for *p* < 0.05; n ≥ 6.

## Additional file

Additional file 1:
**Two-way ANOVA Tables.**

## Abbreviations

Aβ: Amyloid-β
AD: Alzheimer’s disease
apoE: Apolipoprotein E
ApoER2: ApoE receptor 2
BDNF: Brain derived neurotropic factor
CaMK-II: Calcium/calmodulin-dependent protein kinase-II
CREB: CAMP-response element binding protein
EFAD mice: Tg-mice expressing 5xFAD mutations and human *APOE2*, *APOE3*, or *APOE4*
FAD: Familial-AD
h: Human
KO: Knockout
LTP: Long-term potentiation
m: Mouse
min: Minute
MWM: Morris water maze
NMDAR: N-methyl-D-aspartate receptor
oAβ: Oligomeric amyloid-β
p: Phosphorylated
p-CREB: Phosphorylated cAMP-response element binding protein
p-CaMK-II: Phosphorylated calcium/calmodulin-dependent protein kinase-II
PSD95: Postsynaptic density protein 95
Tg: Transgenic
TR: Targeted replacement
5xFAD: Tg mice expressing 5 FAD mutations
5xFAD/*APOE*-KO: 5xFAD mice with m-*APOE*-KO
