# Supplementary material for: APOE4 enhances age-dependent decline in cognitive function by down-regulating an NMDA receptor pathway in EFAD-Tg mice
Source: Mol Neurodegener. 2015 Mar 5;10:7. doi: 10.1186/s13024-015-0002-2 (PMC4391134; doi:10.1186/s13024-015-0002-2)
Supplement: Additional file 1: — Two-way ANOVA Tables. [file 13024_2015_2_MOESM1_ESM.docx]

**Appendix 1: 2-Way ANOVA TABLES**
